# Supplementary material for: Complexome profiling of the Chlamydomonas psb28 mutant reveals TEF5 as an early PSII assembly factor
Source: Plant Cell. 2025 Mar 17;37(6):koaf055. doi: 10.1093/plcell/koaf055 (PMC12164590; doi:10.1093/plcell/koaf055)
Supplement: koaf055_Supplementary_Data [file koaf055_supplementary_data.zip › psb28_interactive.zip!/plots/intensity/qualityPlots/peptides/CGLD27_Cre05.g237050.t1.1.html]

### Cre05.g237050.t1.1

Trivial name: CGLD27  
SeqCoverage [median]: 8.30  
Mass: 31.13 kDa  
  
 

| Sequence | MissedCleavage | Mass [Da] | wtIntensity | muIntensity | Score |
| --- | --- | --- | --- | --- | --- |
| DQRPVNQLAELK | 1 | 1409.76 | 2568.22 | 6254.43 | 109.44 |
| QILTDGVLYSSR | 0 | 1350.71 | 1958.07 | 0.00 | 62.30 |
|  |  |  | 4526.3 | 6254.4 |  |
